# Wavefront analysis and modulation transfer function of three multifocal intraocular lenses

**DOI:** 10.4103/0301-4738.60075

**Published:** 2010

**Authors:** Marcony R Santhiago, Marcelo V Netto, Jackson Barreto, Beatriz AF Gomes, Arthur Schaefer, Newton Kara-Junior

**Affiliations:** 1 Department of Cataract Surgery, University of São Paulo, São Paulo, Brazil; 2 Department of Refractive Surgery, University of São Paulo, São Paulo, Brazil; 3 Department of Ophthalmology, Federal University of Rio de Janeiro, Rio de Janeiro, Brazil

**Keywords:** Cataract surgery, modulation transfer function, multifocal intraocular lenses, wavefront

## Abstract

**Purpose:**

To evaluate wavefront performance and modulation transfer function (MTF) in the human eye after the implantation of diffractive or refractive multifocal intraocular lenses (IOLs).

**Materials and Methods:**

This was a prospective, interventional, comparative, nonrandomized clinical study. Uncorrected distance and near visual acuity, and wavefront analysis including MTF curves (iTrace aberrometer, Tracey Technologies, Houston, TX, USA) were measured in 60 patients after bilateral IOL implantation with 6 months of follow-up. Forty eyes received the diffractive ReSTOR (Alcon), 40 eyes received the refractive ReZoom (Advanced Medical Optics) and 40 eyes, the Tecnis ZM900 (Advanced Medical Optics). The comparison of MTF and aberration between the intraocular lenses was performed using analysis of variance (ANOVA), followed by the Dunn test when necessary.

**Results:**

The mean uncorrected distance visual acuity was similar in all three groups of multifocal IOLs. The ReSTOR group provided better uncorrected near visual acuity than the ReZoom group (*P* < 0.001), but similar to the Tecnis group. Spherical aberration was significantly higher in the ReZoom group (*P* = 0.007). Similar MTF curves were found for the aspheric multifocal IOL Tecnis and the spheric multifocal IOL ReSTOR, and both performed better than the multifocal IOL ReZoom in a 5 mm pupil (*P* < 0.001 at all spatial frequencies).

**Conclusions:**

Diffractive IOLs studied presented similar MTF curves for a 5 mm pupil diameter. Both diffractive IOLs showed similar spherical aberration, which was significantly better with the full-diffractive IOL Tecnis than with the refractive IOL ReZoom.

The intraocular light-scattering and higher order aberrations due to refractive or diffractive optics may lead to a poor retinal image quality and undesirable symptoms such as disability glare, halos, and reduction of contrast sensitivity in patients implanted with multifocal intraocular lenses (IOLs).[1–8]

In order to improve visual quality after cataract removal, IOL materials and designs have been extensively studied worldwide. The multifocal diffractive IOL Tecnis ZM900, based on the Huygens-Fresnel principle, has a prolate aspheric anterior surface which reduces spherical aberrations.[9,10] The multifocal diffractive ReSTOR has a central 3.6-mm apodized optic region where 12 concentric diffractive zones on the anterior surface have a gradual reduction in diffractive step heights from the center to the periphery.[11,12] The IOL ReZoom is a second-generation refractive multifocal IOL and distributes light over five optical zones.[8,13]

Wavefront sensors available now have added quite useful information on the optical quality of the eye. Aberrometers provide an objective measurement of optical aberrations that include sphere, cylinder, and higher order aberrations, such as spherical aberration, coma, trefoil, and other ones. Wavefront sensors also provide image quality metrics such as the modulation transfer function (MTF), which displays the ratio of image contrast to object contrast for ocular optics as a function of the spatial frequency of a sinusoidal grating.[14–18]

The aim of this prospective study was to determine if differences in the multifocal IOL surface and its multifocality would mean differences in wavefront data, mainly higher order aberration, such as coma and spherical aberration, and contrast transferred analyzed with MTF between an aspherical multifocal diffractive IOL (Tecnis), a spheric apodized diffractive multifocal IOL (ReSTOR), and a refractive multifocal IOL ReZoom.

## Materials and Methods

This prospective interventional comparative clinical study enrolled 120 eyes of 60 patients subjected to bilateral multifocal IOL implantation. It should be noted that the surgery occurred on different days with an interval of at least 2 weeks between eyes of the same patient. The study was approved by the institutional review board. Patients were enrolled consecutively in a nonrandomized fashion, as previous studies with multifocal IOLs, based on the clinical judgment of the investigators, to receive either an aspheric multifocal IOL Tecnis ZM900 (40 eyes; Advanced Medical Optics, Inc.), a spheric multifocal IOL AcrySof ReSTOR SN60D3 (40 eyes; Alcon Laboratories, Rochester, NY, USA), or a multifocal IOL ReZoom (40 eyes; Advanced Medical Optics, Santa Ana, CA, USA).[4,8] Because of design differences in the IOL's surfaces, the choice of IOL was not randomized in this study. Instead, the investigator selected the multifocal IOL he or she believed would most accurately meet the individual patient's needs in terms of function and tolerability. The purpose of this study was not to compare visual acuity and performance in daily activities; instead, the purpose was to compare objective parameters which could represent differences in the optical quality of eyes implanted with different multifocal IOLs with different designs. Besides that, to minimize the bias of the study we tried to have a higher number of subjects, compared to previous ones. Informed consent was obtained and the study was in adherence to the tenets of the Declaration of Helsinki.

Patients with a bilateral visually significant cataract with corneal astigmatism lower than 1.0 D (diopters) were eligible for inclusion in the study. Exclusion criteria were any ocular diseases, such as corneal opacities or irregularity, dry eye, amblyopia, anisometropia, glaucoma, retinal abnormality, surgical complications, IOL tilt, IOL decentration greater than 0.4 mm (estimated by retroillumination), or incomplete follow-up.

Patients were examined before surgery and at 1, 7, 30, 90 and 180 days after surgery. At 180 days postoperatively, uncorrected distance (4 m), and uncorrected near (0.4 m) visual acuities (UCVA) were measured as well as higher order aberration values and the MTF curve.

Visual acuity was measured using the early treatment of diabetic retinopathy study charts under photopic conditions (target luminance of 90 cd/m^2^). The visual acuity values were converted to the logarithm of the minimal angle resolution scale (logMAR) for statistical analysis and then presented in a decimal scale. All eyes were targeted for emmetropia.

Wavefront analysis and modulation transfer function curve were obtained using the *i*Trace aberrometer (Tracey Technologies, Houston, TX, USA) which uses ray-tracing technology to obtain wavefront data. All aberrations were measured up to the sixth Zernike order. MTF curves were obtained considering the mean value for each spatial frequency of each IOL. The MTF studied refers to distance vision.

Measurements were repeated three times to obtain a well-focused aligned image of the eye. Measurements were taken for the maximum pupil diameter and then analyzed for 5.0 mm pupils. Pupils were dilated with two drops of cyclopentolate 1% given 15 min apart. Measurements were taken 45 min after the last cyclopentolate drop was instilled. The pupil diameter was measured using the Colvard pupillometer (Oasis Medical, Glendora, CA, USA). Five millimeters was chosen as the maximum diameter analyzed in order to avoid misinterpretation of the centroids resulting from the size of the capsulorrhexis and the edge of the IOL. Besides that, with 5 mm we can have an appropriate wavefront analysis with the aberrometer device.

All surgeries were performed by one experienced surgeon with standardized small-incision phacoemulsification with IOL implantation in the capsular bag. Continuous curvilinear capsulorrhexis with an approximate 5.0 mm diameter was created. No adverse event has occurred.

Statistical analysis was performed using SPSS for Windows (version 11.5, SPSS, Chicago, IL, USA). We had 40 eyes in each group. A sample size of 40 patients per group (120 in total) would allow an effect size of 0.25. The sample sizes took into account a significance level of 5% and a power of 90% for the ANOVA test. All statistical tests were conducted at an alpha level of 0.05. The analysis of age and IOL power was performed using analysis of variance (ANOVA) and the differences were calculated using the multiple comparison Tukey test. The analysis of uncorrected visual acuity for far and near distance was performed using the Kruskal-Wallis test. The X^2^ test was used to verify the proportion between men and women. The comparison of MTF and aberration between the IOLs was performed using analysis of variance (ANOVA), followed by posthoc Dunn test when necessary.

## Results

One hundred and twenty eyes of 60 patients (26 men [43.3%] and 34 women [56.7%]) with a mean age of 62.02 ± 9.09 years were enrolled in this study. No significant difference was found between groups for mean age (*P* = 0.820) and IOL power (*P* = 0.923). Twenty patients received Tecnis in one eye and Restor in the fellow eye; 10 patients received Tecnis in one eye and ReZoom in the fellow eye; 10 patients received ReSTOR in one eye and ReZoom in the fellow eye; 10 patients received ReZoom in both eyes; 5 patients received ReSTOR in both eyes; 5 patients received Tecnis in both eyes. Most frequent choice was two diffractive IOLs in the same patient because we believe that would provide a pseudoacommodative amplitude that would match that patient's needs.[4,6–8,11,12]

There were no intraoperative complications in any of the eye. At 6 months postoperatively, all the lenses were well centered and there was no evidence of posterior capsule opacification. There were no problems to complete the follow-up with any patient.

At 6 months postoperatively, all eyes showed improvement in UCVA. The spherical equivalent was 0.21 ± 0.17 in the Tecnis group, 0.21 ± 0.17 in the ReSTOR group, and 0.20 ± 0.18 in the ReZoom group (*P* = 0.840). The mean distance UCVA was 0.74 ± 0.20 in the ReSTOR group, 0.80 ± 0.19 in the ReZoom group, and 0.76 ± 0.22 in the Tecnis group. There was no significant difference among the three IOL groups for distance UCVA (*P* = 0.286). The mean near UCVA was 0.96 ± 0.10 in the ReSTOR group, 0.83 ± 0.19 in the ReZoom group, and 0.93 ± 0.14 in the Tecnis group. Considering near UCVA, the ReSTOR group provided better performance than ReZoom group (*P* < 0.001). No significant difference was found between ReSTOR and Tecnis (*P* = 0.963) and between Tecnis and ReZoom (*P* = 0.156).

Postoperative wavefront analysis revealed [Table 1] total aberration root-mean-square (RMS) values of 0.89 ± 0.74 (m (Tecnis), 1.30 ± 1.30 (m (ReSTOR), and 2.13 ± 2.16 (m (ReZoom). No statistically significant difference was found between ReSTOR and Tecnis groups (*P* = 0.435). The performances of both ReSTOR and Tecnis IOLs were better than ReZoom (*P* = 0.001). Mean postoperative higher order aberration values were 0.58 ± 0.72 (m (Tecnis), 0.92 ± 1.14 (m (ReSTOR), and 1.27 ± 2.06 (m (ReZoom). No statistically significant difference was found between the three IOL groups (*P* = 0.094).

**Table 1:** Wavefront analysis for a 5 mm pupil

	Mean (μm) ± SD			
	
	Total RMS	HOA RMS	Coma RMS	Spherical aberration RMS
Tecnis	0.89 ± 0.74	0.58 ± 0.72	0.21 ± 0.22	0.06 ± 0.05
ReZoom	2.13 ± 2.16	1.27 ± 2.06	0.50 ± 0.72	0.27 ± 0.47
ReSTOR	1.30 ± 1.30	0.92 ± 1.14	0.35 ± 0.52	0.15 ± 0.18
*P* value	0.001*	0.094	0.060	0.007*

HOA - total higher order aberration; RMS - root mean square; SD - standard deviation.

* Statistically significant difference between groups (ANOVA)

When analyzing higher order aberrations separately, coma values were 0.21 ± 0.22 (m (Tecnis), 0.35 ± 0.52 (m (ReSTOR), and 0.50 ± 0.72 (m (ReZoom). No statistically significant difference was found between the three IOL groups (*P* = 0.060). Spherical aberration values were 0.06 ± 0.05 (m (Tecnis), 0.15 ± 0.18 (m (ReSTOR), and 0.27 ± 0.47 (m (ReZoom). The Tecnis IOL obtained significant lower values of spherical aberration when compared to ReZoom IOL (*P* = 0.005). No statistically significant difference was found between ReSTOR and Tecnis (*P* = 0.351) and between ReSTOR and ReZoom (*P* = 0.164) [Table 1].

**Figure 1:**
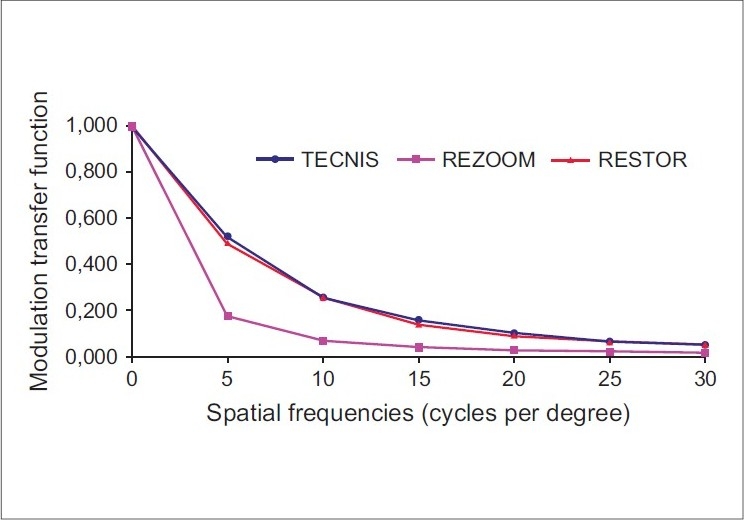
Modulation transfer function curves according to IOL groups for the 5 mm pupil diameter at different spatial frequencies. The modulation transfer function curve shows the contrast transferred at different spatial frequency for each IOL. The aspheric multifocal IOL Tecnis performed similar to spheric multifocal IOL ReSTOR. And both performed better than multifocal IOL ReZoom in a 5 mm pupil (*P* < 0.05). The MTF does refer to distance vision

**Figure 2:**
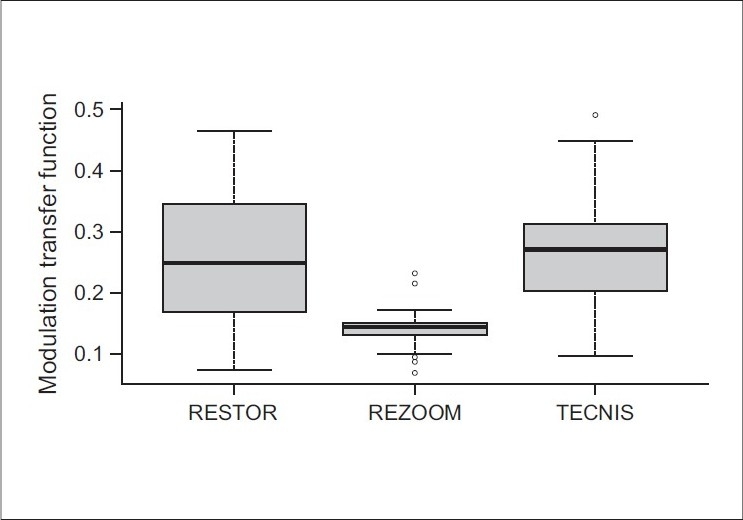
Box plot showing the average height from the modulation transfer function curve according to IOL groups. The aberrometer device used in this study gives an average height for each MTF curve. This box plot shows the average height and the difference among three multifocal IOLs, considering the mean modulation transfer ratio at the spatial frequencies evaluated in the study. The contrast transferred was statistically similar in the Tecnis and ReSTOR group. For a 5 mm pupil diameter, the ReZoom group transferred less contrast (*P* < 0.05)

Postoperative MTF curves were obtained for each IOL group. No statistically significant difference was found between Tecnis and ReSTOR for all spatial frequencies. Both Tecnis and ReSTOR IOLs had a superior performance than ReZoom IOL (*P* < 0.001 at all spatial frequencies) for a 5 mm pupil diameter [Fig. 1]. The *i*tracey aberrometer software used to define the MTF curve also provides the average height for each MTF curve studied. This is an average modulation between 0 and 30 cycles/degree [Fig. 2]. The mean MTF value for each multifocal IOL was 0.270 ± 0.097 (Tecnis), 0.255 ± 0.112 (ReSTOR) and 0.109 ± 0.025 (ReZoom). No statistically significant difference was found between Tecnis and ReSTOR (*P* = 0.709). Both Tecnis and ReSTOR IOLs had significantly higher values than ReZoom for a 5 mm pupil diameter (*P* < 0.001).

## Discussion

New IOL designs, with modified prolate surfaces, intend to reduce the total amount of spherical aberration in the eye, favoring visual quality.[18–21] However, combination of far, intermediate, and near visual correction with an acceptable optical quality is quite challenging.[22,23] In this study, the intraocular optical quality of the multifocal IOLs was compared using wavefront analysis and MTF curves were analyzed with 5.0 mm pupil diameters.

In our study, a low spherical aberration error was observed in the aspheric IOL compared to a conventional spherical IOL after 6 months, as previously reported by other authors[18–21,24,25]; however, it was not statistically significant (*P* = 0.351) [Table 1].

The spheric ReSTOR IOL tends to increase the spherical aberration levels due to its less prolate periphery comparing to the Tecnis aspherical design. Nevertheless, the apodized diffractive surface of the ReSTOR IOL probably behaves as an aspheric surface. It progressively decreases the height of the diffractive steps from the center to the periphery, showing less spherical aberration.[11,12,26–29]

The diffractive IOL Tecnis had statistically significant lower values of spherical aberration compared to refractive IOL ReZoom [Table 1]. In patients with the multifocal IOL ReZoom, these data could mean reduced contrast sensitivity. Cillino *et al*.[6] showed similar performance for both the Tecnis group and ReZoom group in contrast sensitivity tests except at three cycles per degree.

Traditionally, we measure wavefront aberrations to relate them to optical quality.[16–21] However, with multifocal lenses, controlled amounts of aberration are desired to give near vision. Differences we see in spherical aberration could also be due to more multifocality and not only to the asphericity of the lenses. With ReZoom, the power oscillates rapidly between two values. To fit this wavefront shape, lots of spherical aberration, not just of fourth order, but also sixth and eighth would be needed to represent it.

In the present study, no statistically significant difference in the spherical aberration error was found between ReSTOR and ReZoom in a 5 mm pupil analysis. Ortiz *et al*.[28] showed that the ReZoom group had higher spherical aberration values than the ReSTOR group with a 5.0 mm pupil (*P* = 0.003). According to the authors, the ReSTOR IOL was the least affected by the pupil diameter due to its apodized design, which is similar to that of an optical filter.

In our study, the mean value of spherical aberration (0.15 ± 0.18 μm) of multifocal ReSTOR was higher, compared to other studies such as Toto *et al*.'s[26] (0.13 ± 0.04 μm analyzed 6 months after surgery), Zelichowska *et al*.'s[29] (0.09 ± 0.00 μm analyzed 6 months after surgery), Souza *et al*.'s[12] (0.09 ± 0.05 μm analyzed 3 months after surgery), and Rocha *et al*.'s[27] (0.09 ± 0.05 μm analyzed 2 months after surgery). Although all of them used a 5 mm pupil diameter for data analysis, they used different aberrometers and therefore different technologies to obtain the wavefront data.

The use of wavefront sensors on diffractive lenses is quite challenging.

Diffractive IOLs by their very nature create discontinuities in the wavefront to create multifocal wavefronts. Wavefront sensors such as the iTrace used here measure the wavefront slope, and then integrate to get the ocular wavefront. However, integrating a function results in an extra constant term. Commercial wavefront sensors, such as the iTrace device, must assume that this constant provides a smooth and continuous wavefront; thus the data should be read with caution.

Although we found results similar to other studies with a ray tracing technology, Charman *et al*.[30] showed Hartmann-Shack aberrometers that use longer wavelengths of infrared light are more likely to produce satisfactory results for eyes with diffractive IOLs.

Coma aberrations provide information on whether the IOL is properly centered. In our study, the mean value of coma-like aberration was lower in the Tecnis group compared to the ReSTOR group and ReZoom group, even though the difference found was not statistically significant (*P* = 0.060). Dietze *et al*.[31] suggested that correcting spherical aberration with aspheric IOLs could produce more coma aberration when the IOL is decentered. It leads us to conclude, indirectly, that our lower rates of decentration could justify the lower levels of coma aberration error in the Tecnis group. However, a longer follow-up would be more appropriate to analyze because an asymmetric contraction of a fibrotic capsule could develop and decenter the IOL.[32–34]

The MTF defines how optical systems (the IOL in this study) modulate contrast sensitivity as a function of spatial frequency. Comparing the multifocal diffractive IOLs, the aspheric multifocal IOL Tecnis performed similarly to spheric multifocal IOL ReSTOR.

In our study, the ReZoom group had statistically significant worse values for each spatial frequency than the other two types of multifocal IOLs (*P* < 0.001). However, these data should be read with caution. A limitation of this study was that the MTF measurements were done with only a 5.0 mm pupil.[35,36] However, we decided to study 5.0 mm because we can have an appropriate wavefront analyzes with an aberrometer device. In further studies, the MTF measurements could be done at varying pupil sizes, since this would give a more realistic evaluation of daily living.

Other limitation of this study is that the aberration data analyzed did not consider internal aberration separately. With no statistically significance difference in corneal aberration between the IOL groups, it would be possible to have precise information about the influence of the multifocal IOL in the aberrations of the eye. Besides that, the Tracy device performs monochromatic measurements, the results of which cannot be compared to the human polychromatic vision.

In conclusion, despite the difficulty to obtain wavefront data for eyes implanted with multifocal IOLs, the *in vivo* assessment of multifocal IOL performance with wavefront sensors certainly provides useful objective information about each IOL's optical quality. In our study, the contrast transferred for a 5 mm pupil diameter was similar for diffractive IOLs, Tecnis ZM900, and ReSTOR. Wavefront assessment showed lower values of spherical aberration with the multifocal IOL Tecnis even though they statistically significant only when compared to the refractive ReZoom.
